# Influences of body mass index and physical activity on hypertension and stroke in Korean adult males: 10-year longitudinal study

**DOI:** 10.20463/jenb.2017.0003

**Published:** 2017-06-30

**Authors:** Yonghwan Kim, Haemi Jee

**Affiliations:** 1. Sports Health Medicine Center, Asan Medical Center, Seoul Republic of Korea; 2. Department of Sports and Health Care, Namseoul University, Cheonan Republic of Korea

**Keywords:** Hypertension, Stroke, Physical activity, Obesity, Longitudinal study

## Abstract

**[Purpose]:**

Routinely performed health screening results of 5,624,503 Korean men between ages 20 to 70 obtained from the National Health Insurance Service (2002-2013) were assessed for this study. Data of subjects who met the initial criteria were divided into three groups based on their BMI: normal weight (18.5 to <25.0 kg/m2), overweight (25.0 to <30.0 kg/m2), and obese (≥30.0 kg/m2) groups. The results were further sub-divided by physical activity frequencies (days/week). The disease codes for hypertension and stroke were provided by the National Health Insurance Service for the adjusted relative risks (RR) assessment with the Cox proportional hazard model.

**[Methods]:**

Routinely performed health screening results of 5,624,503 Korean men between ages 20 to 70 obtained from the National Health Insurance Service (2002-2013) were assessed for this study. Data of subjects who met the initial criteria were divided into three groups based on their BMI: normal weight (18.5 to <25.0 kg/m^2^), overweight (25.0 to <30.0 kg/m^2^), and obese (≥30.0 kg/ m^2^) groups. The results were further sub-divided by physical activity frequencies (days/ week). The disease codes for hypertension and stroke were provided by the National Health Insurance Service for the adjusted relative risks (RR) assessment with the Cox proportional hazard model.

**[Results]:**

Significant RRs of hypertension and stroke were shown in the overweight moderately active group (3 – 4 days/week). In addition, significant RR of hypertension was shown in the normal weight moderately active group. No significance was seen in the obese group in all physical activity frequencies.

**[Conclusion]:**

Regularly performed moderate amount of physical activity may be beneficial in reducing the risk for hypertension and stroke. However, the effects of excessive body weight may override the positive effects of physical activity on the occurrence of hypertension and stroke.

## INTRODUCTION

Hypertension and stroke have been some of the major causes of mortality, promoting prominent social, economic, personal, and emotional burdens in Korea^1,2^. Prominent causes of these adult diseases have been related to factors such as sex, sedentary lifestyle, obesity, high caloric diet, alcohol consumption, and smoking^3,4^. Among the major factors, obesity and physical inactivity have been reported as strong determining factors for promoting occurrence of hypertension and stroke^5^. These rapidly rising diseases may be largely prevented through proper lifestyle management that includes proper nutrition, body weight, alcohol consumption, smoking, and physical activity^6^. 

The American College of Sports Medicine (ACSM) recommends 5 days or more per week of moderate to vigorous intensity physical activity with maintenance of a body mass index (BMI) of 25 kg/m^2^ or less for healthy lifestyle management^7^. However, the number of people who fulfill such recommended amount of physical activity, including 3 days or more of walking, is far less than expected. Although physical activity is one of the vital controllable factors for managing blood pressure and prevention of various adult diseases, participation and long-term adherence rates are rather low^8,9^. For example, according to a report by the Ministry of Health & Welfare, less than 50% of people fulfill the recommended amount of physical activity^7,10^. The adherence rates in some of the European countries ranged from 52% to 82%, indicating significant influential factors in physical activity participation such as various political and public health interventions^9^. A study that analyzed factors for physical activity adherence reported that intensive public awareness efforts may promote adherence to the regular performance of physical activity^9^. 

Moreover, a rapidly increasing number of IOT (Internet of things)- based appliances and lifestyle devices with increased use of automobiles tend to promote a sedentary lifestyle and reduction in everyday activities. In addition, increased tendency for intellectual and sedentary tasks have prevented sparing time and space for physical activity. Such phenomena influencing increased sedentary lifestyles have been occurring worldwide, including developed and developing countries. With a rapid increase in economic development and aged population, Korea has been facing age-related diseases such as hypertension and stroke. The World Health Organization (WHO) has been recognizing problems associated with such chronic diseases and their related mortality rates. WHO stated that about 60% of deaths globally are related to chronic disease and the organization is taking measures to prevent chronic disease-related mortality. Relative to such concerns, worldwide research has been conducted in an effort to elucidate and prevent chronic diseases such as hypertension and stroke^11^. 

Worldwide, many longitudinal studies have been conducted to determine effective chronic disease prevention and related mortality and morbidity. However, long-term longitudinal studies to elucidate measures to prevent chronic diseases have been poorly conducted in Korea. Therefore, this study aimed to analyze a nationally collected large-scale data set to elucidate the occurrence of hypertension and stroke in relationship to BMI and physical activity. The results of the study may clarify the role of physical fitness in the health of adult Korean males and may further explain the role of physical activity in hypertension and stroke via a 10-year longitudinal study. 

## METHODS

### Study subjects and procedure

The study sample compromised 5,624,503 Korean male adults between the ages of 20 and 70 who had participated in the 2002, 2003, 2012, and 2013 health screening by the Korean National Health Insurance Service. Health screening has been conducted biannually by the National Health Insurance Service in extension of the regularly performed health examination. Data of those who participated in the 2002 and 2003 health screening results were used as a baseline to match and track reexamined data in the years 2012 and 2013. Subjects with other chronic diseases or those of other nationalities were also excluded from the study. Through the elimination process, this study analyzed health screening results of 10 years to find and collect data of 7,549,450 subjects between the ages 20 to 70. Data with missing physical activity data were also excluded to obtain data of 5,624,503 subjects. BMI was used as a division criteria for dividing the subjects into normal weight (18.5 to ≤25.0 kg/m2), overweight (25.0 to ≤30.0kg/m2), and obese (>30.0 kg/m2) groups.

### Physical activity, alcohol consumption, and smoking

The questionnaires on physical activity, alcohol consumption, and smoking were self-administered. There were two alcohol related open-ended questions: “how many times do you drink a week?” and “how much do you drink per day?” The respondents answered the questions by writing down the number of days per week or drinks by glass. The WHO risk criteria were used for the health screening and the study. There was one multiple choice question on physical activity with five response options: “how many times (or days) a week do you exercise until you feel sweat?” and the answers were (1) none, (2) 1 – 2 days, (3) 3 – 4 days, (4) 5 – 6 days, and (5) almost daily. Responses were then subdivided into 3 groups: 1) inactive (0 – 2 days of physical activity), 2) moderately active (3 – 4 days of physical activity), and 3) highly active (5 – 7 days of physical activity). Three questions were asked on smoking: 1) “are you currently smoking or do you have a history of smoking?”, 2) “how many cigarettes per day?”, and 3) “how long have you been smoking?” The first of the smoking questions offered a multiple-choice response of with (1) never smoked, (2) previously smoked but quit, (3) currently smoking. Possible answers for other questions were similar to that of physical activity.

### Selected diseases

This study targeted the adult diseases of hypertension and stroke. Possible occurrence of either disease was identified with the disease codes provided by the National Health Insurance Service for those who have submitted claims for medical care and benefits. The disease codes are the codes used by the Korea Center for Disease Control (KCDC) for categorizing various diseases for insurance and official purposes. The codes for stroke and hypertension are I60-I69 and I10-I15, respectively. 

### Statistical analysis

SAS (version 9.4; SAS Institute Inc. Cary, NC, USA) was used for all statistical analyses with a p-value of <0.05 set for significance in this study. Means and standard deviations were calculated for continuous variables and one-way ANOVA was conducted to observe the differences between groups. As for the categorical data, Chisquare test was conducted to observe the group differences after percent calculations. A significance level of 0.05 was used for the analysis. Relative risk (RR) analysis was conducted through the Cox proportional hazard model to obtain occurrence risks of the diseases in relations to BMI. Age and health habits such as alcohol consumption and smoking were used as correction factors. BMI was used again as a correction factor during calculation for RR in the obese group due to the wide range. 

## RESULTS

### General characteristics

General characteristics of the study subjects are organized in Table 1. The subjects were first categorized into three groups (A, B, C) based on BMI and the amount of physical activity as previous mentioned. Mean ages with standard deviations of the normal weight, overweight, and obese groups were 43.4±14.1, 43.8±12.2, and 40.0±11.7 years, respectively, with significant differences. Total cholesterol (TG), fasting plasma glucose (FPG), aspartate aminotransferase (AST), and alanine aminotransferase (ALT) levels significantly increased with increase in BMI. The TG levels were 189.1±46.9 IU/L and 209.0±48.6 IU/L and the FPG levels were 95.0±31.2 mg/dL and 101.8±35.2 mg/dL, respectively for the normal weight and obese groups (p<0.00). The overall physical activity participation rates were very low for all groups. Among the study subjects, about 10.1 to 12.1% participated in a moderate amount of physical activity (3 – 4 days per week), 7.8 to 7.9% participated in a high amount of physical activity (5 or more days per week), indicating low rates of physical activity. As for smoking, 49.8%, 45.4%, and 51.0% of the normal weight, overweight, and obese groups were current smokers, respectively. The obese group had the greatest number of current smokers. In addition, the obese group had the least amount of subjects with previous history of smoking, 34.8%, in comparison to normal weight and overweight groups of 36.3% and 38.3%, respectively. Lastly, the normal weight group showed the highest rate and the obese group showed the lowest rate of alcohol consumption, 5.5% and 4.1% respectively. 

**Table 1.** Physiological and anthropometric characteristics of the subjects.

Variables	Normal weight	Overweight	Obese	p-value
BMI (kg/m^2^)	18.5 - <25	25.0 - <30	≥30.0	
Subjects, n(%)	3,790,383 (67.4)	1,691,802 (30.1)	142,318 (2.5)	
Age (years)	43.4 ± 14.1	43.8 ± 12.2	40.0 ± 11.7	<0.001
Height (cm)	169.3 ± 6.3	169.5 ± 6.1	170.5 ± 6.3	<0.001
Weight (kg)	63.8 ± 7.2	76.8 ± 6.7	92.2 ± 9.0	<0.001
BMI (kg/cm^2^)	22.2 ± 1.9	26.7 ± 1.2	31.6 ± 1.8	<0.001
TC (mg/dl)	189.1 ± 46.9	202.9 ± 49.3	209.0 ± 48.6	<0.001
Glucose (mg/dl)	95.0 ± 31.2	98.7 ± 32.1	101.8 ± 35.2	<0.001
AST (IU/L)	27.0 ± 23.4	30.1 ± 21.3	36.4 ± 25.3	<0.001
ALT (IU/L)	26.5 ± 26.4	37.0 ± 32.1	52.8 ± 44.5	<0.001
Physical Activity (%)				
Inactive group	82.1	79.2	80.7	<0.001
Moderately active group	10.0	12.1	11.5	
Highly active group	7.9	8.7	7.8	
Smoking (%)				
Never	36.3	38.3	34.8	<0.001
Quit	13.9	16.3	14.2	
Current	49.8	45.4	51.0	
Alcohol (%)				
None	56.2	52.7	52.5	<0.001
Low risk	28.2	30.7	31.3	
Moderate risk	10.1	11.8	12.1	
High risk	5.5	4.8	4.1	

Normal weight: BMI, 18.5 –<25.0 kg/m^2^, Overweight: BMI, 25.0 - <30.0 kg/m^2^, Obese: BMI, ≥30.0 kg/m^2^, BMI: body mass index, AST: aspartate transaminase, ALT: alanine transaminase, TC: total cholesterol, Inactive group: 0 - 2 days per week, moderately active group: 3 - 4 days per week, highly active group: 5 – 7 days per week, p-value: <0.05

**Table 2.** Adjusted relative risk of hypertension according to BMI and physical activity.

Groups	Frequency (d/wk)	Subjects (n)	Incidence rate (%)	Relative ratio (95% CI)	p-value
Normal weight	0 - 2	462,065	14.8	1	
	3 - 4	56,186	14.8	0.99 (0.99 - 1.00)	0.24
	5 - 7	49,963	18.9	0.99 (0.99 - 1.01)	0.94
Overweight	0 - 2	325,598	23.8	1	
	3 - 4	48,646	24.0	0.99 (0.98 - 0.99)	<0.001
	5 - 7	36,022	28.2	0.99 (0.99 - 1.00)	0.17
Obese	0 - 2	34,122	29.4	1	
	3 - 4	4,908	30.6	1.01 (0.99 - 1.04)	0.26
	5 - 7	3,044	31.7	0.98 (0.95 - 1.01)	0.25

Normal weight: BMI, 18.5 –<25.0 kg/m^2^, Overweight: BMI, 25.0 - <30.0 kg/m^2^, Obese: BMI, ≥30.0 kg/m^2^, BMI: body mass index, Frequency (d/wk): days of physical activity performed expressed in days per week, CI: confidence interval, Relative ratio: relative risk adjusted by age, smoking, and alcohol.

**Table 3.** Adjusted relative risk of stroke according to BMI and physical activity.

Groups	Frequency (d/wk)	Subjects (n)	Incidence rate (%)	Relative ratio (95% CI)	p-value
Normal weight	0 - 2	116,332	3.7	1	
	3 - 4	12,900	3.4	0.96 (0.95 - 0.98)	<0.001
	5 - 7	13,610	5.1	0.99 (0.98 - 1.02)	0.85
Overweight	0 - 2	55,818	4.1	1	
	3 - 4	7,937	3.9	0.96 (0.94 - 0.98)	<0.001
	5 - 7	7,105	5.6	0.99 (0.97 - 1.02)	0.74
Obese	0 - 2	3,855	3.3	1	
	3 - 4	570	3.6	1.03 (0.95 - 1.13)	0.46
	5 - 7	435	4.5	1.05 (0.95 - 1.16)	0.31

Normal weight: BMI, 18.5 –<25.0 kg/m^2^, Overweight: BMI, 25.0_-_<30.0 kg/m^2^, Obese: BMI, ≥30.0 kg/m^2^, BMI: body mass index, Frequency (d/wk): days of physical activity performed expressed in days per week, CI: confidence interval, Relative ratio: relative risk adjusted by age, smoking, and alcohol.

### Adjusted relative risk (RR) of hypertension based on BMI and physical activity

Relative risk (RR) of hypertension according to BMI and physical activity after adjustment for age, smoking, and alcohol consumption did not show significant differences based on physical activity. However, the moderately active group showed significant RR of 0.99 (95% CI=0.98~0.99, p=0.00) in comparison to the inactive group, without significant RR in the overweight group. The highly active group did not show significance (p=0.17). 

### Adjusted relative risk (RR) of stroke based on BMI and physical activity

Relative risk of stroke based on BMI and physical activity after adjustment for age, smoking, and alcohol consumption showed significant RR in the moderately active group in comparison to the inactive group 0.96 (95% CI=0.95-0.98, p<0.01) in the normal weight group. Significant RR of 0.96 (95% CI=0.94-0.98, p<0.01) was shown in the moderately active group in comparison to the inactive group in the normal weight group. In the overweight group, the moderately active group showed significant RR of 0.96 (95% CI=0.94-0.98, p<0.01) in comparison to the inactive group in the overweight group. In the obese group, the moderately active group (p=0.46) and highly active group (p=0.31) did not show significant RR. In addition, both normal weight and overweight groups did not show significant RR in the highly active group in comparison to the inactive group. 

## DISCUSSION

This study was conducted to observe the differences in occurrence of hypertension and stroke based on the degree of obesity and the amount of physical activity in Korean males aged between 20 and 70 using 10 years of longitudinal data. The occurrence rates of hypertension in adult males and females over 20 years of age have been reported to be 32.2% and 25.4%, respectively, according to the Ministry of Health & Welfare^10^. Prevention measures for hypertension and stroke have been associated with regularly performed physical activity and weight reduction with management of cholesterol and glycemic level^7^. 

Overweight or obesity is known to occur through imbalance of energy consumption and energy expenditure for the accumulation of excess calories. Such energy accumulation has been reported to be preventable with increased physical activity. Despite the emphasis on the importance of physical activity for prevention of various adult diseases including hypertension and stroke, the performance rate of physical activity to the level recommended by internationally acknowledged organizations such as the American Heart Association (AHA), the World Health Organization (WHO), and the ACSM (American College of Sports Medicine) have been insufficiently low^7,11,12^. 

Promotion of the proper amount of physical activity should be emphasized through knowledge and properly conducted research. Despite the importance of elucidating the relationship between regular performance of physical activity and adult diseases, longitudinal studies have been rare and limited in Korea. The limiting factors for conducting a longitudinal study may be related to the amount of time and money involved with conducting cross-national studies. Therefore, this study was conducted using the biannually administered KNHANES, a nationally recognized government organization. KNHANES is a trans-national health-related survey of the general Korean population providing comparatively unbiased results for a population based analysis. This survey also has the advantage of regularly and repeatedly performed results obtained in a clinically-based assessment.

The AHA recommends lifestyle modification and the blending of regularly performed weight management through increased fiber consumption, low fat diet, low sodium consumption, low alcohol consumption, and regularly performed physical activity^13^. Most of the research reports positive effects of exercise and physical activity, especially aerobic exercise. A study that conducted a treadmill exercise program with 24 subjects for 8 to 12 weeks reported a significant reduction in systolic blood pressure by 6±12 mmHg and diastolic blood pressure by 3±7 mmHg by the end of the study^14^. In addition, a study which conducted a meta-analysis of 54 randomized controlled trials (RCT) reported that properly conducted physical activity had significant systolic blood pressure and diastolic blood pressure reduction effects by 3.84 mmHg and 2.58 mmHg, respectively^15^. 

Multitudinal epidemiological studies have also reported positive results on the relationship between regularly performed physical activity and blood pressure^16- 18^. A meta-analysis study that analyzed 13 different research studies from a total follow-up of 15,607 subjects, out of 136,846 subjects, for the assessment of alteration in blood pressure reported significant adjusted RR of 0.81 with recreational level of physical activity, and adjusted RR of 0.89 with moderate intensity physical activity^19^. Such results were similar to those obtained through this study. Despite similar findings, results of this study showed no significant relationship between high blood pressure and increased frequency of physical activity in the highly active group. However, some previous studies reported mixed results on the relationship between physical activity and the prevalence of hypertension^19, 20^. In addition, ACSM reported a possible lack of relevance on the evidence for an inverse dose-response relationship between physical activity and hypertension. Moreover, as for the categorization of evidence for the occurrence of hypertension, ACSM categorized physical activity in the ‘B’ category, which suggests a lesser or comparatively weaker relationship between hypertension and physical activity^7^. The reason for such a comparatively feeble relationship has been explained by the number of strong influential risk factors for hypertension. In addition, it is difficult to isolate physical activity as a sole factor in relationship to blood pressure in a longitudinal study that may last for years^21^. 

There are also several evidence-based and review studies that indicate that the frequency and intensity of physical activity may not always lead to reduction in blood pressure. A meta-analysis of 13 prospective cohort studies with 136,846 persons reported an inverse dose-response association between levels of recreational physical activity and risk of hypertension, whereas there was no significant association between occupational physical activity and hypertension^18^. Recently, several studies reported increased hypertension with excessive physical activity. Exercise is generally known to improve blood pressure through the possible influence of nitric oxide (NO) and reduction in endothelin-1, a potent vasoconstrictor peptide, produced by vascular endothelial cells^22^. However, continuously increased blood flow and mechanical stress on the endothelial wall of the artery during prolonged physical activity may lead to arterial stiffness and exercise induced hypertension^23, 24^. The Eighth Joint National Committee (JNC) guideline on the management of adult hypertension advised 3 to 4 physical activity sessions per week lasting 40 minutes per session in the form of aerobic exercise^25^. The committee report also endorsed the Lifestyle Work Group report that based its report on the recommendations of the AHA and the American College of Cardiology^25^. Such reports coincide with the findings of this study that moderately active groups showed significantly less relative risk for the occurrence of hypertension. 

Age adjusted risk factor analysis for the occurrence of stroke reported that 90.5% of the stroke burden was attributable to modifiable risk factors, including behavioral factors such as smoking, diet, and sedentary lifestyle^4^. In addition, strong association between high systolic blood pressure and intracerebral hemorrhage (hazard ratio 1.4495% CI 1.32–1.58), subarachnoid hemorrhage (1·431·25–1·63), and stable angina (1.411.36–1.46) were reported through a prospective cohort study^3^. Stroke accounts for 1 per every 10 deaths with 1 occurrence of stroke every 5 minutes in Korea. In addition, stroke attacks 213 males and 220 females per 100,000 persons a year, with an increase in the number of victims with increased age^2^. 

Because stroke has similar risk factors as hypertension, regular performance of physical activity has been reported to have a preventative effect^26^. However, since the prevalence rate increases with age, physical activity as a sole influencing factor may show limited results despite adjustments for other influencing factors. Statistical adjustment has a limited role in elucidating the cause-and-effect relationship between physical activity and stroke. Therefore, ACSM’s guideline categorized physical activity as a comparatively low influential factor in the category of evidence for the occurrence of stroke^27^. Despite difficulties in elucidating the causeand- effect relationship between stroke and physical activity, a meta-analysis study reported 25% and 17% reductions in the prevalence rate of stroke, in subjects who participated in high level physical activity and moderate level physical activity, respectively based on 22 cohort studies^28^. According to a study conducted with Caucasian and African Americans in the U.S., a 33% increase in the rate of stroke was shown in those who did not participate in physical activity week^29^. However, a significant difference was not observed between the subjects who participated in physical activity more than 4 days a week and less than 4 days a week^29^. In this study, a significant relationship between physical activity and occurrence of stroke was shown in those who participated in a moderate amount of physical activity (3 to 4 days per week). Such a significant relationship was not shown in those who participated in the high level of physical activity (5 days or more per week). 

In addition, the obese groups did not show a significant RR for stroke nor hypertension in all frequencies of physical activity in this study. Several reasons could be suspected for such results. First, the influence of body weight may have been a comparatively greater influential factor than physical activity. According to the JNC 7 report, the range of blood pressure reduction through physical activity was between 4 to 9 mmHg whereas the range of blood pressure reduction through weight reduction was 5 to 20 mmHg, suggesting greater influence of body weight on blood pressure^12^. Moreover, a largescale study reported that physical activity had limited influence on the risk for atrial fibrillation occurrence in males with excess body weight^30^. A meta-analysis based on 97 prospective cohorts indicated that about half of the excess risk of coronary heart disease and three-quarters of the excess risk of stroke was associated with high BMI^31^. Previous reports support the finding of the study that regularly performed moderate amount of physical activity with proper weight management is vital in avoiding adult diseases, stroke, and hypertension. 

There may be several reasons for observing a significant relationship between a moderate amount of physical activity and occurrence of stroke and hypertension. First, it is difficult to determine the appropriate amount of exercise required for maximally improving vascular health and CVD outcomes. For example, a study that combined Taiwanese, American, and European population studies to assess the dose-response relationship between physical activity and CVD mortality reported maximal risk reduction at 41 METs h/week^32^. Although greater amount or volume of physical activity did not correlate with adverse results, the morality rate seemed to increase slightly as the volume increased with a wide range of confidence interval for variation in results^32-34^. Similar findings can be found for hypertension. Although appropriate amounts of physical activity on most days have been recommended to lower blood pressure, excessive amounts of physical activity have been reported to increase blood pressure^18,23,24^. Such observations may be partially explained by variation in perceived intensity efforts. A study which observed the degree of perceived exercise intensity according to physical activity intensity reported that individuals underestimated exercise intensity as the intensity increased^33,34^. 

There are several limitations to this study. This study was a 10-year retrospective study with one time tested assessment results. Physical activity and BMI levels between the tested years could not be included in the consideration of factors. Since habits such as physical activity, smoking, and alcohol consumption may change on a monthly or yearly basis, such information could have further explained the cause-and-effect relationship. Such factors should be considered in future studies. Moreover, the data was strictly controlled by the National Health Insurance Service to protect the confidentiality of the public. Since the focus was on providing better assessment and results with data protection, there were limitations in study design, data collection, and data analysis. Therefore, a limited amount of information could be provided through this study. In addition, considering that the prevalence rate of adult diseases has been known to begin in the mid-40s age range, grouping by age group may further allow understanding of the diseases in relationship with physical activity. In addition, physical activity should further be categorized by duration, frequency, intensity, and type of exercise according to the ACSM’s guideline for exercise prescription. 

## CONCLUSION

A 10-year longitudinal analysis on the relative risk of hypertension and stroke based on BMI and frequency of physical activity of Korean male adults indicated the hypertension and stroke risk lowering effects of moderately performed physical activity of 3 to 4 days per week in those with a BMI between 25.0 and <30 kg/m^2^. Moderately performed physical activity also had a hypertension risk lowering effect in those with BMI between 18.5 and <25.0 kg/m^2^. Obesity may be a strong influential factor that may mitigate the effects of physical activity. 
